# Prevalence and predictors of physical inactivity levels among Kenyan adults (18–69 years): an analysis of STEPS survey 2015

**DOI:** 10.1186/s12889-018-6059-4

**Published:** 2018-11-07

**Authors:** Muthoni Gichu, Gershim Asiki, Pamela Juma, Joseph Kibachio, Catherine Kyobutungi, Elijah Ogola

**Affiliations:** 1 Division of Non Communicable Diseases, Ministry of Health, Nairobi, Kenya; 2 The Institute of Global Health, Faculty of Medicine, University of Geneva (UNIGE), Geneva, Switzerland; 3 African Population and Health Research Center, Nairobi, Kenya; 4 Clinical Medicine, University of Nairobi, Nairobi, Kenya

**Keywords:** Physical inactivity, Non-communicable diseases, Kenya

## Abstract

**Background:**

Physical inactivity accounts for more than 3 million deaths worldwide, and is implicated in causing 6% of coronary heart diseases, 7% of diabetes, and 10% of colon or breast cancer. Globally, research has shown that modifying four commonly shared risky behaviours, including poor nutrition, tobacco use, harmful use of alcohol, and physical inactivity, can reduce occurrence of non-communicable diseases (NCDs). Risk factor surveillance through population-based periodic surveys, has been identified as an effective strategy to inform public health interventions in NCD control. The stepwise approach to surveillance (STEPS) survey is one such initiative, and Kenya carried out its first survey in 2015. This study sought to describe the physical inactivity risk factors from the findings of the Kenya STEPS survey.

**Methods:**

This study employed countrywide representative survey administered between April and June 2015. A three stage cluster sampling design was used to select clusters, households and eligible individuals. All adults between 18 and 69 years in selected households were eligible. Data on demographic, behavioural, and biochemical characteristics were collected. Prevalence of physical inactivity was computed. Logistic regression used to explore factors associated with physical inactivity.

**Results:**

A total of 4500 individuals consented to participate from eligible 6000 households. The mean age was 40.5 (39.9–41.1) years, with 51.3% of the respondents being female. Overall 346 (7.7%) of respondents were classified as physically inactive. Physical inactivity was associated with female gender, middle age (30–49 years), and increasing level of education, increasing wealth index and low levels of High Density Lipoproteins (HDL).

**Conclusion:**

A modest prevalence of physical inactivity slightly higher than in neighbouring countries was found in this study. Gender, age, education level and wealth index are evident areas that predict physical inactivity which can be focused on to develop programs that would work towards reducing physical inactivity among adults in Kenya.

## Background

Physical inactivity has been identified as the fourth leading risk factor for global mortality causing approximately 3 million preventable deaths worldwide [1]. Epidemiological research shows that 15–20% of the overall risk for coronary heart disease, type 2 diabetes, colon cancer, breast cancer is due to physical inactivity, which also leads to fractured hips in older persons [2]. Physical inactivity causes 6% of the burden of disease from coronary heart disease, 30% of ischemic heart disease, 7% of type 2 diabetes, 10% of breast cancer, and 10% of colon cancer [3, 4]. Examination of meta-analyses, systematic reviews, and pooled analyses performed by the 2018 Physical Activity Guidelines Advisory Committee of the US Department of Health and Human Services confirms significant health benefits due to physical activity for different non-communicable diseases (NCDs) [5].

The economic consequences of physical inactivity on healthcare cost are substantial mainly due to indirect costs such as the value of reduced economic output because of illness, disease related work disabilities and premature death [6–9]. Mortality rates from NCDs increase with increasing body weight, and are markedly increased when individuals are classified as obese [10]. Regular physical activity is a protective factor against unhealthy weight gain [5, 10]. For these reasons, the World Health Organization (WHO) passed resolution WHA55.23 in 2002 [11] and formulated a global strategy to address physical activity and health in 2004 [12]. Subsequently, a 10% relative reduction in prevalence of insufficient physical activity was set as one of nine goals to be met by 2020 in the Global Action Plan 2013–2020 for NCDs [13].

Regrettably, ‘physical activity transition’ referring to resultant decline in physical activity levels, coupled with increasing sedentary behaviours over time [14, 15], is affecting younger people, including children and the youth, leading to negative health consequences such as obesity particularly among urban residents [16–18]. According to the Global School Health Survey of 2003 conducted among students aged 13 to 15 years in Kenya, only 11.1% met the required regular physical activity threshold of at least 60 min per day and up 40.9% were reported to have sedentary habits [19]. The 2016 Kenya Report Card indicated that only half of Kenyan children and adolescents were engaging in sufficient levels of physical activity [20]. Although these surveys used different methods, the low levels may have contributed to the observed increase in overweight and obesity among the children with 4% of rural and 21% of Kenyan urban children being overweight or obese [21]. The increase has been associated with demographic and social changes such as globalization, urbanization, aging population and adoption of unhealthy lifestyles [22] that is especially relevant to developing countries like Kenya.

Recognizing the importance of NCDs, the Ministry of Health in Kenya published the National Strategy for the Prevention and Control of NCDs for 2015–2020 [23]. While this strategy mentions promotion of physical activity as one of the objectives where in there are no specific policies and interventions in place to address the situation despite the declining levels of physical activity, and the associated increase in obesity and overweight among the adults and children [23]. Moreover, there is limited population-based data to inform the much needed policies and interventions. The STEPs survey conducted in 2015 generated national level data on NCDs risk factors including physical inactivity thus provided an opportunity to profile population level factors to inform policy towards the reduction of NCDs as well as promote physical activity.

## Methods

We use data from the STEPs Kenya 2015 survey, a nationally representative household survey conducted between April and June 2015 as detailed elsewhere [24].

Data on physical activity was collected using the Global Physical Activity Questionnaire (GPAQ) as a self-report [25]. This tool was developed by the WHO, and has been validated for physical activity surveillance in developing countries. Physical activity was determined from the amount of time being physically active in three domains; transport, at work and during leisure time. Through a self-report questionnaire participants were asked about the intensity of their physical activity (vigorous or moderate) guided by a show card (a visual aid demonstrating various activities by intensity) for work and leisure, inclusion of ten continuous minutes spent walking or cycling for transport, number of days they performed these activities in a typical week and amount of time spent on each activity on a typical day [24]. Data collection was administered using a Personal Digital Assistant (PDA) loaded with eSTEPS software. The system was designed to allow PDA to show only a question and all its possible answers at a time in one screen view, and also provided various filters, skips and validation procedures.

### Statistical analysis

In this study, physical inactivity was defined as obtaining less than 150 min of moderate intensity physical activity throughout the week or less than 75 min of vigorous intensity physical activity throughout the week or less than an equivalent combination of moderate- and vigorous intensity derived from WHO definition of physical activity [11]. The proportion of participants who met this definition was estimated overall and according to a variety of participant characteristics. The independent variables used in this study were in three categories: demographic (sex, age, marital status, highest level of education, occupation, residence, wealth status), behavioural (tobacco use and alcohol consumption), and physical and biochemical measurements (waist circumference, blood pressure and cholesterol). Men with waist circumference of > 104 cm or women with waist circumference of 88 cm were classified as centrally obese, Waist hip ratio of 0.85 among women and 0.90 men was considered as obesity risk. Systolic blood pressure > 140 and diastolic pressure > 90 were categorized as hypertension. Low HDL was defined as < 1.03 mmol/L among men and < 1.29 mmol/L among women.

Logistic regression analyses were used to compute adjusted odds ratios for each exposure variable while controlling for all the other variables (confounders) in the model. *P*-values of less than 0.05 were considered significant. The analysis was done using STATA software, version 14.

## Results

### Characteristics of study participants

In total 6000 adults were invited to participate in this study, 4500 (75%) completed the survey and 4484 (74.4%) had no missing data. Table 1 presents the characteristics of the sample. The study participants were adults aged 18 to 69 years with a mean age of 40.5 (39.9–41.1) years, with slightly fewer males than females (48.7% versus 51.3%). Most respondents (> 85%) had some education (mainly secondary), were rural residents (62%), employed (80%) and married (65%).

**Table 1:** Demographic characteristics and physical inactivity prevalence among adults in the Kenya STEPS survey 2015

Characteristics	Total	(%)	Physically Inactive (%)
Sex			
Male	2186	48.75	175 (8.0)
Female	2298	51.25	172 (7.5)
Age groups			
18–29	2062	45.99	193 (9.4)
30–39	1045	23.31	57 (5.5)
40–49	695	15.50	37 (5.4)
50–59	443	9.88	32 (7.3)
60–69	239	5.33	26 (10.9)
Education level			
No formal education	563	12.56	70 (12.5)
Primary incomplete	1044	23.28	59 (5.7)
Primary complete	1000	22.30	62 (6.2)
Secondary and above	1877	41.86	155 (8.2)
Residence			
Rural	2776	61.91	167 (6.0)
Urban	1708	38.09	179 (10.5)
Occupation			
Unemployed	493	10.99	54 (10.9)
Employed	936	20.87	80 (8.6)
Homemaker	981	21.88	81 (8.3)
Non-paid/volunteer	19	0.42	2 (10.7)
Self-employed	1749	39.01	90 (5.2)
Student	306	6.82	39 (12.7)
Marital status			
Not married	1039	23.17	103 (10)
Married	2938	65.52	200 (6.8)
Formerly married	507	11.31	43 (8.5)

### Prevalence of physical inactivity

A total of 346 (7.7%) of the respondents were classified as physically inactive. Physical inactivity was more prevalent among those in middle age (30–49 years), among those with no formal education, in the urban areas, among students, non-paid/− volunteers, unemployed, and single adults (Table 1).

### Factors associated with physical inactivity

Results from the multivariable analysis indicated women were 1.72 times more likely to be physically inactive than men. Physical inactivity was twofold higher among those aged 30–39 years, and 40–49 years compared to those aged 50–69 years, and was 2–5 odds higher among those with education compared to those with no formal education. Individuals self-reporting the middle wealth quintile were three times more likely to be physically inactive than those in the poorest and second wealth quintile. Individuals with low level of HDL had double odds of being physically inactive. Being in the richest wealth quintile had lower odds (0.39) of physical inactivity compared to those within the lowest level of wealth quintile. There was borderline association of physical inactivity with rural residence (OR = 1.61 95% CI [0.96, 2.69], central obesity (OR = 1.53 95% CI [0.99, 2.35]) and lower odds with hypertension (OR = 0.67, 95% CI (0.44, 1.02) - Table 2.

**Table 2:** Predictors of physical inactivity among adults aged 18–69 years in Kenya (2015)

Predictor	Adjusted Odds Ratio (95% CI)	p-value
Gender		
Male	1.00	
Female	1.72 (1.15, 2.56)	0.008*
Age group		
50–69	1.00	
18–29	1.17 (0.70, 1.98)	0.550
30–39	2.04 (1.12, 3.73)	0.021*
40–49	1.90 (1.01, 3.61)	0.048*
Residence		
Urban	1.00	
Rural	1.61 (0.96, 2.69)	0.069
Education level		
No formal education	1.00	
Primary incomplete	2.54 (1.38, 4.67)	0.003*
Primary complete	5.33 (2.54, 11.19)	0.000*
Secondary and above	2.99 (1.60, 5.60)	0.001*
Wealth band		
Poorest	1.00	
Second	1.23 (0.63, 2.39)	0.550
Middle	2.94 (1.15, 7.54)	0.025*
Fourth	0.86 (0.42, 1.75)	0.672
Richest	0.39 (0.18, 0.84)	0.016*
Tobacco use		
No	1.00	
Yes	1.32 (0.75, 2.34)	0.334
Weekly alcohol drinking		
No alcohol	1.00	
Daily	1.97 (0.47, 8.23)	0.351
3 or more days	0.80 (0.36, 1.78)	0.581
Less than 3 days	0.93 (0.60, 1.45)	0.753
Hypertension		
No	1.00	
Yes	0.67 (0.44, 1.02)	0.064
Waist circumference		
Normal	1.00	
Centrally obese	0.83 (0.52, 1.31)	0.417
Waist Hip Ratio		
Normal	1.00	
Centrally obese	1.53 (0.99, 2.35)	0.055
HDL cholesterol		
Normal	1.00	
Low	1.83 (1.09, 3.08)	0.023*

*Significant at *p* < 0.05

## Discussion

Using data from a nationally representative survey in Kenya, the prevalence of physical inactivity was found to be 7.7%, similar to that reported in other sub-Saharan Africa (SSA) countries such as Burkina Faso (7.8%), Malawi (8.4%) and Ghana (8.8%) [26]. This was significantly higher than in the neighbouring country, Uganda with 4.3% prevalence of physical inactivity reported in the recent STEPS survey [27]. South Africa (44.7%), Mauritania (52.6%) and Swaziland (49.1%) reported higher prevalence of physical inactivity [26]. These results support the notion that prevalence of physical inactivity is a function of country’s income and is usually high in developed countries and low in less developed and underdeveloped countries [28]. However, this differs from the STEPs surveys undertaken in same countries with differing prevalence, Burkina Faso (17.7%), Malawi (9.5%), Ghana (85.7%), Mauritania (50.7%) and Swaziland (15.3%) [29]. This could be attributed to the differences in the methodology used between the STEPs survey and the studies. The estimated global adult physical inactivity prevalence in 2008 was 31% [29]. Although the prevalence of physical inactivity is not alarming in Kenya yet, it is likely to increase without timely policy put in place. Factors associated with physical inactivity were identified.

In the Kenyan population the factors significantly associated with physical inactivity in the analyses undertaken include: female gender, middle age 30–49 years, formal education, middle wealth and low levels of HDL.

Similar to a number of other studies [30–34], women were more likely to be physically inactive than men in this study. To a large extent, men tend to engage more in vigorous and moderately intense sports activities than women (83.7% versus 16.3%) and this could be attributed to social-cultural factors including gender roles. More specifically, these factors include the dominance of work and family responsibilities on women, social norms that permit higher levels of PA in men especially in work domains, lack of social support for women to be active, social isolation, environmental constraints, economics, and low levels of personal knowledge and motivation that limit physical activity among women [35–37].

In this study nearly two thirds of the sample classified as physically inactive were middle aged adults between 30 and 49 years. Micklesfield et al. made a similar observation in South Africa where 43–50% of physically inactive were young adults [38]. Young people in SSA engage more in light and incidental moderate-intensity physical activity during domestic chores and active transportation while the older people make conscious decisions to exercise for health benefits [39]. This finding is not surprising given the potential increased risk perception of NCDs among older people which may lead to greater interest in engaging in preventive measures such as physical activity.

Contrary to observations by Benjamin and Linda in the United States of America (USA) [40], physical inactivity increased with level of education in our study. This finding could be explained as a result of the low priority accorded to physical activities in schools as revealed in a study among Kenyan school children in 2014, which showed that children were spending a considerable amount of time sitting in class with light intensity physical activity due to more emphasis on grades compromising the quality and quantity of physical education and most children used motorised transport to and from school [41].

Being in the middle wealth quintile was associated with higher levels of physical inactivity similar to what was observed in Vietnam [34]. This could be due to physical inactivity arising from using motorized transport and a more sedentary work environment for the middle class. Individuals in the richest wealth quintile were less physically inactive than the other wealth categories; this may be due to their additional resources to access and utilize leisure time physical activity opportunities.

Physical inactivity was associated with low levels of HDL similar to other research [42]. This result was to be expected as physical activity is known to increase levels of HDL [42–44].

There were borderline associations between physical inactivity and rural residence, and between physical inactivity and central obesity. The association of physical inactivity with rural residence in Kenya is surprising because previous publications indicated physical inactivity to be higher among urban than rural residents in SSA [44–47]. The Kenyan situation here was in agreement with the observations by Wilcox et al. in USA [48], where rural residents were less physically active compared to urban residents. This is explained by limited opportunities for leisure related physical activities in the rural areas despite the engagement in physical activity in other domains of work and transport. The borderline association between physical inactivity and central obesity also observed in our study is consistent with the report of Kruger et al. in South Africa, however there may be other contributing factors like the difference in development [49].

Low- and middle-income countries such as Kenya contribute almost three quarters of global NCD deaths, 82% of all NCD premature deaths (below age of 70 years) [10, 50] and the highest number of health years lost/disability adjusted life years worldwide [51]. With the projected rise in the burden of NCDs in low- and middle-income settings [10, 50], addressing physical inactivity which is a major risk factor for NCDs in these settings will be crucial in meeting the global target of 10% relative reduction in the prevalence of insufficient physical activity [13]. In Kenya, the objectives promote physical activity stated in the National Strategy for the Prevention and Control of NCDs for 2015–2020 [23] should be operationalized towards this global goal.

This study had some limitations. In the STEPwise survey physical activity was self-reported. Previous studies on the validity of self-reported measure of physical activity suggest that reliability of self-report is dependent on factors such as the type of questionnaire used, participants’ age and the type, duration and intensity of physical activities measured by self-reporting [52, 53]. In addition, there could be a variance in the validity of the self-report due to ethnicity, language, education and health status [54–56]. However, the Global Physical Activity Questionnaire used in the WHO STEPwise survey has been validated and found suitable and acceptable instrument for monitoring physical activity in population health surveillance systems in developing countries [25].

## Conclusion

This study has for the first time reported the country-wide prevalence of physical inactivity among adults aged 18–64 years in Kenya and identified population groups to be targeted for interventions. In order to increase physical activity at population level and thus reduce physical inactivity, interventions should be targeted to women, people within middle age, the middle class, and to post-secondary students in schools. Physical activity in Kenya which is mostly work related is more often than not viewed as a means to have work completed and the link to health is missing. There is need to keep in mind that this self-report may be subjective. Among the four major risk factors for NCDs, physical inactivity is the only risk factor with no policy for promotion of physical activity. This paper provides relevant information for health policymakers to establish interventions to increase physical activity levels among risk groups identified as well as among the general population.

## Abbreviations

HDL: High Density Lipoproteins
NCDs: Non-Communicable Diseases
SSA: Sub-Saharan Africa
USA: United States of America
WHO: World Health Organisation
